# Single-Trial Detection and Classification of Event-Related Optical Signals for a Brain–Computer Interface Application

**DOI:** 10.3390/bioengineering11080781

**Published:** 2024-08-01

**Authors:** Nicole Chiou, Mehmet Günal, Sanmi Koyejo, David Perpetuini, Antonio Maria Chiarelli, Kathy A. Low, Monica Fabiani, Gabriele Gratton

**Affiliations:** 1 Department of Computer Science, Stanford University, Stanford, CA 94305, USA; sanmi@cs.stanford.edu; 2 Beckman Institute for Advanced Science and Technology, University of Illinois Urbana, Champaign, Urbana, IL 61801, USA; mehmet@illinois.edu (M.G.); lowka@illinois.edu (K.A.L.); mfabiani@illinois.edu (M.F.); grattong@illinois.edu (G.G.); 3 Department of Engineering and Geology, “G. D’Annunzio University” of Chieti-Pescara, 65127 Pescara, Italy; david.perpetuini@unich.it; 4 Department of Neuroscience, Imaging and Clinical Sciences, “G. D’Annunzio University” of Chieti-Pescara, 66100 Chieti, Italy; antonio.chiarelli@unich.it; 5 Institute for Advanced Biomedical Technologies, “G. D’Annunzio University” of Chieti-Pescara, 66100 Chieti, Italy; 6 Psychology Department, University of Illinois Urbana, Champaign, Champaign, IL 61820, USA

**Keywords:** fast optical signals (FOS), event-related optical signals (EROS), brain–computer interface (BCI), machine learning (ML), deep learning

## Abstract

Event-related optical signals (EROS) measure fast modulations in the brain’s optical properties related to neuronal activity. EROS offer a high spatial and temporal resolution and can be used for brain–computer interface (BCI) applications. However, the ability to classify single-trial EROS remains unexplored. This study evaluates the performance of neural network methods for single-trial classification of motor response-related EROS. EROS activity was obtained from a high-density recording montage covering the motor cortex during a two-choice reaction time task involving responses with the left or right hand. This study utilized a convolutional neural network (CNN) approach to extract spatiotemporal features from EROS data and perform classification of left and right motor responses. Subject-specific classifiers trained on EROS phase data outperformed those trained on intensity data, reaching an average single-trial classification accuracy of around 63%. Removing low-frequency noise from intensity data is critical for achieving discriminative classification results with this measure. Our results indicate that deep learning with high-spatial-resolution signals, such as EROS, can be successfully applied to single-trial classifications.

## 1. Introduction

Brain–computer interface (BCI) refers to methods that enable direct communication between the brain and a computer system without reliance on other effectors (e.g., muscles). These systems typically employ sensors that measure brain activity, which is then processed through a classification system enabling one to infer the intention of the human involved with some degree of confidence. Ideally, BCIs should transmit information rapidly, commonly measured in bits per minute (b/m). Human speech, in comparison, is transmitted at approximately 2400 b/m across various languages [1].

Besides high information transmission rates, practical sensor systems for BCI applications should also be non-invasive, inexpensive, and portable. It is not surprising, therefore, that most of the current BCI research and developments exploit measurements of the scalp electroencephalographic (EEG) activity. However, EEG-based BCIs have limited speed, largely arising from the challenge of distinguishing among more than a handful of brain states at any moment due to the low spatial resolution of electrical signals measured at the scalp.

Gratton et al. [2] pursued an alternative approach called Event-Related Optical Signal (EROS) to study brain activity. EROS measures changes in near-infrared light scattering associated with neuronal depolarization and hyperpolarization. These changes, if occurring in superficial areas of the cortex, can be measured from the surface of the head using devices similar to those used for functional near-infrared spectroscopy (fNIRS). This measurement takes the form of changes in the amount of light reaching a detector (“intensity”) or changes in the photons’ time-of-flight (“phase delay” when measured by frequency-domain optical systems). EROS signals possess high spatial resolution (of the order of 1 cm or less) and temporal resolution (of the order of 100 ms or less) [3]. Such a combination of spatial and temporal resolutions offers the potential for the non-invasive measurement of dozens of signals from the human brain in parallel every 100 ms, yielding theoretical information transmission rates of thousands of b/m. However, a significant problem is that EROS has a low signal-to-noise ratio (SNR) [4]. Therefore, practically all reports of brain activity measured with EROS until now have been based on averages across multiple trials (≥15) [5,6,7]. Relying on averaging across trials to improve reliability would correspondingly slow down the communication transmission rate of a hypothetical EROS-based BCI. It should be noted, though, that averaging is a naive procedure for increasing SNR; other more sophisticated methods might be more efficient.

This study aims to classify individual trials based on EROS measures of brain activity. A data set from a previously published study in which participants responded to stimuli using either their left or right hand was used [8]. EROS data were recorded from four different, but overlapping, montages covering large portions of the scalp, including regions over the left and right motor areas. The current study focused on brain activity immediately preceding and following each motor response to determine whether the EROS measures collected during this interval could accurately predict whether the participant would produce a right or left-hand response on a given trial.

To enhance discrimination between response types, this study applied machine learning techniques. Machine learning algorithms have revolutionized data analysis across various scientific disciplines, enabling pattern recognition from historical data for classification, regression, and clustering tasks. However, classical machine learning methods, such as support vector machines (SVMs), random forests (RFs), decision trees (DTs), and k-nearest neighbors (k-NN), typically require manual feature extraction and selection. These methods rely heavily on domain expertise and/or a priori knowledge about the data of interest to pre-process data and extract relevant features. These features are then used as input to train the machine learning model.

In contrast, deep learning, a subset of machine learning, has gained prominence for its ability to learn features from raw data without any *a priori* feature selection. For example, it has demonstrated success in the domains of medical image analysis [9,10], physiological time series [11], electronic health records (EHR) [12,13], and wearable sensors [14,15,16]. Recent advances in deep learning for BCI applications have reduced reliance on manual feature extraction. While many BCI systems still use handcrafted features [17,18,19], deep learning offers significant advantages. Manual identification of relevant features across populations may not generalize to individual subjects, potentially excluding relevant subject-specific features. Additionally, extracting handcrafted features tailored to each subject is not a scalable process.

Deep learning with convolutional neural networks (CNNs) has shown exceptional performance in image and signal processing tasks due to their ability to optimize and apply filters, or convolutional kernels, through automated learning. Combined with their hierarchical structure, this automatic feature learning capability enables CNNs to efficiently capture spatial and temporal dependencies in natural signals, often outperforming classical machine learning methods in complex data scenarios. However, these models require a large amount of training data to learn such correlations, involve a large number of tunable hyperparameters (e.g., filter kernel size, optimization step size), and are challenging to interpret.

Despite these challenges, CNNs demonstrate high adaptability across various applications. While CNNs have been widely investigated for natural image processing tasks [20,21], their usage in BCI applications is relatively nascent. Therefore, the potential usage of CNNs for processing spatiotemporal signals such as EEG and EROS warrants domain-specific consideration and a rigorous understanding of designing and training such models. Much of recent work has focused on EEG-based BCI, yielding studies exploring the application of CNNs to various BCI applications, including motor imagery classification [22,23,24,25,26,27], detection of visual-evoked responses [28,29,30,31], and epilepsy prediction and monitoring [32,33,34,35,36]. Notably, a paradigm-agnostic CNN utilizing depthwise separable convolutional layers has shown robust performance across diverse EEG-based BCI tasks [37]. This approach is grounded in well-known EEG feature extraction concepts, such as optimal spatial filtering and filter-bank construction. In addition, depthwise and separable convolutions enhance parameter efficiency compared to standard convolutions, enabling the model to perform well under limited data settings [38,39,40]. Additionally, this CNN architecture generates a spatiotemporal summary of the input signal, facilitating the post hoc identification of discriminative signal portions that contribute to the model’s classification.

Traditionally, EEG has been the primary modality for BCI systems, with research applying classical and deep learning techniques to classify EEG data. Although EROS offers high spatial and temporal resolutions, most prior works relied on averages across multiple trials to address EROS’ low SNR. Furthermore, the literature remains divided on whether intensity or phase delay measurements are more effective for EROS classification [7,41]. Additionally, the investigation of machine learning approaches to EROS has been limited to classical machine learning classifiers coupled with domain-specific features [7,41]. For instance, Proulx et al. [7] utilized SVM and Linear Discriminant Analysis (LDA) with EROS (intensity and phase delay), achieving a 63.6% classification accuracy using multi-trial (≥15) averaging in a visual oddball task. Another study employed SVM for single-trial analysis of EROS, reporting a 63.0% accuracy for retinotopy classification [41].

To date, the potential of deep learning for enhancing single-trial EROS classification remains unexplored. This study addresses this gap by leveraging CNNs to automatically extract complex spatial and temporal relationships from single-trial EROS data in a limited subject-specific data regime.

In summary, this study investigated the feasibility of using a convolutional neural network (CNN) architecture to distinguish single-trial event-related optical signal (EROS) activity associated with different behavioral outputs. Additionally, it aimed to determine whether EROS measures obtained via intensity or phase delay offer better discrimination. This distinction is crucial for future instrument design, as phase delay measures necessitate a more complex frequency-domain (FD) recording system, while intensity measures can be achieved with a simpler continuous-wave (CW) recording system. Finally, this study explores different CNN training paradigms to examine the effectiveness of using subject-specific data from multiple recording montages.

**Contributions:**

- Conducting a subject-specific evaluation of CNNs for single-trial classification of EROS data.
- Comparing the discriminative potential of phase versus intensity data.
- Exploring various subject-specific model training paradigms using data from multiple recording montages.
- Analyzing the impact of data quality on classification performance.
- Performing a post hoc investigation of relevant time intervals and spatial locations contributing to the model’s predictions.

The paper is organized as follows: Section 2 introduces the related literature on machine learning for BCI systems. Section 3 discusses the details of data collection, signal pre-processing, and machine learning methodology. Section 4 presents the results, compares them with existing work, and interprets learned neural network features. Section 5 discusses the results in the context of brain–computer interface (BCI) systems, and Section 6 summarizes the conclusions of the work.

## 2. Related Work

Table 1 presents a summary of related works employing classical machine learning and deep learning algorithms for EEG- and EROS-based BCI applications.

### 2.1. Machine Learning Classification Algorithms for EEG

Machine learning techniques have been applied to the classification of EEG signals, often relying on fixed feature extraction methods to compare the performance of different classifiers. For motor imagery (MI) classification, band power features derived from spectral signal representations are commonly used.

In their study, Herman et al. [17] performed a comparative analysis of various methods for quantifying the frequency content of EEG recordings within an MI framework. They evaluated four feature extraction approaches: spectral estimation, atomic decompositions, quadratic time–frequency distributions, and wavelet-based techniques. Their findings indicated that discriminative frequency bands and optimal feature extraction methods vary among subjects, highlighting the potential advantage of automated end-to-end feature learning. Nonetheless, across all participants, power spectral density (PSD) methods proved more robust for extracting MI-related EEG spectral patterns. Furthermore, the study showed that linear methods, such as linear discriminant analysis (LDA) and regularized Fisher discriminant (RFD), offered better intersession generalizability in offline settings. While the applicability of spectral methods to continuous classification in online experiments remains unexplored, the authors suggest using a moving window method for future EEG activity analysis.

Other studies have focused on extensive comparisons of classification methods alongside task-specific EEG feature extraction. Wang et al. [18] examined the classification accuracy of LDA, quadratic discriminant analysis (QDA), kernel Fisher discriminant (KFD), support vector machine (SVM), multilayer perceptron (MLP), learning vector quantization (LVQ) neural network, k-nearest neighbor (k-NN), and decision tree (DT) across two different EEG datasets. They found that all methods achieved satisfactory accuracy (>70%) and emphasized the importance of regularization and dimensionality reduction for nonlinear methods. This study highlights the impact of feature extraction on the optimal classification algorithm and advocates for an end-to-end approach to feature extraction and classification. It also points out that extracting features for motor imagery and execution tasks requires significant domain-specific knowledge.

Similarly, Zhang et al. [19] compared various LDA variants, including their proposed Z-score LDA method, using the common spatial pattern (CSP) algorithm to select optimal spatial filters for EEG signal transformation and feature extraction.

### 2.2. Convolutional Neural Networks for EEG

Offline-based feature extraction methods focus on static energy features, often overlooking the discriminative temporal information within the signal recording. Conversely, CNNs excel at learning these temporal features from raw or pre-processed recordings by applying convolutional kernels along the time dimension.

Several studies have employed techniques that transform the EEG signal into an image representation before applying the CNN. For instance, Tabar and Halici [22] utilized a short-time Fourier transform (STFT) on 2-s segments (500 samples) to convert each EEG channel into a 2D image (x-axis: time, y-axis: frequency), aggregating along the channel dimension to form a 3D tensor. This approach ignores precise spatial information, though a 4D input tensor could incorporate 2D spatial locations. Similarly, Olivas-Padilla and Chacon-Murguia [23] proposed an algorithm that uses CSP to select discriminative frequency bands using an SVM. This method transforms the raw EEG signal in 2-s intervals with the chosen spatial filter but requires separate spatial filters for each pairwise class combination, reducing scalability for large multi-class problems. Miao et al. [24] leveraged domain expertise to determine the time intervals and frequency ranges for processing, decomposing the EEG signal into ten discrete subbands, which might limit the ability to identify trends across broader, continuous frequency ranges.

Other studies have bypassed extensive domain-specific pre-processing, opting for end-to-end feature learning by applying CNNs directly to raw EEG signals. Zhang et al. [25] introduced a novel EEG-inception architecture that uses multiple inception and residual modules, inspired by computer vision, to classify subject-specific motor imagery EEG (MI-EEG) with minimal pre-processing. They also conducted an ablation study on the effects of data augmentation and neural network depth on classification performance, demonstrating comparable performance with a lower standard deviation than state-of-the-art methods.

Similarly, Schirrmeister et al. [26] compared CNN architectures against the filter bank common spatial patterns (FBCSPs) baseline for motor decoding from EEG recordings, showing comparable performance. They detailed deep learning techniques that enhance CNN performance for EEG-based BCIs with minimal pre-processing, including regularization schemes and data augmentation, and provided insights into using pre-trained model weights to address dataset size limitations. Unlike the architecture from Schirrmeister et al. [26], which relies on data augmentation for training a large parameter count CNN, Lawhern et al. [37] proposed a parameter-efficient depthwise separable CNN that performs well across multiple BCI tasks and surpasses previous methods in limited data settings. They compared their model’s performance against the best traditional models for each BCI paradigm, evaluated both within-subject and cross-subject classification, and investigated CNN feature explainability.

### 2.3. EROS-Based BCI

EROS offers high spatial and temporal resolution, making it suitable for detecting localized brain activity. However, noninvasive recording of event-related optical signals is a challenging task due to the low SNR. Although independent component analysis (ICA) has been utilized to mitigate noise and global interference in event-related fast optical signals, the application of machine learning techniques for EROS-based BCIs remains under-explored.

Traditionally, researchers have relied on event-related averaging across multiple trials to enhance SNR [5,6]. For instance, Medvedev et al. [6] demonstrated a significant correlation between independent components of pre-processed single-trial EROS and EEG recordings by simultaneously capturing brain activity with both modalities. More recently, Proulx et al. [7] investigated EROS for BCI applications, focusing on classification reliability during a visual oddball task. They employed fifteen-trial averages and classifiers such as SVM and LDA, achieving an average balanced accuracy of approximately 62–63%. Additionally, Perpetuini et al. [41] used a frequency-domain optical system and SVM to classify visual-field quadrant stimulation, obtaining an above-chance classification accuracy with the highest accuracy of ≈63% using DC light intensity. Despite these advances, the effectiveness of intensity versus phase delay measurements for EROS classification remains debated [7,41].

## 3. Materials and Methods

The methods and data used in this study were derived from a prior study [8] and were tailored to the particular objectives of the deep learning approach utilized for the single-trial detection and classification of event-related optical signals for a BCI. Readers are encouraged to consult the methods section of that manuscript for further details. The analyses included in this manuscript are entirely novel, and none has been previously published.

### 3.1. Participants

Data from 12 right-handed, healthy individuals (7 Female, 5 Male; mean age: 22 years, range: 18–28 years) were included to develop the deep learning models. All participants were native English speakers, with normal hearing, normal to corrected-to-normal vision, and normal speech. All participants provided written informed consent, and the study was approved by the University of Illinois at Urbana-Champaign and conducted accordingly with the ethical standards of the Helsinki Declaration.

### 3.2. Behavioral Task

A detailed description of the entire experimental procedure can be found in Baniqued et al. [8]. Each trial began with a bimodal auditory–visual cue “V” or “H” presented simultaneously on a computer monitor and via speakers for 400 msec. The letter “H” precue indicated a manual (hand) response using the left or right hand. The letter “V” precue indicated a vocal response to be made through a voice key by saying the word “left” or “right”, but these trials were not included in the development of the deep learning model and will not be discussed further. The precue was followed by the reaction stimulus (2000 ms later). The reaction stimulus (duration 400 ms) was either an “L” or “R” presented on the screen or via speakers, each indicating a “left” or “right” response, respectively. Participants responded with a key press using either their left or right index finger. Participants completed 20 blocks of 24 trials during each recording montage, with half of the trials in each block requiring manual responses (“H” precues). This resulted in a maximum of 240 trials used to train the classifier for each montage.

### 3.3. Optical Imaging Recording

Each participant’s optical data were recorded using two independent Imagent frequency domain oximeters (ISS, Inc.; Champaign, IL, USA). Laser diodes emitted near-infrared light (830 nm) modulated at 110 MHz over frontal and central brain regions and 300 MHz for parietal and occipital regions. Prior research showed that these two modulation frequencies yield relatively similar EROS responses when the phase delay data are transformed into picoseconds [42]. To avoid cross-talk between the two systems, sources from one system were never closer than 6 cm from any detector on the other system. To achieve this, recordings were obtained from frontal and parietal regions in one set of runs, and from central and occipital regions in another set. The order of these runs was counterbalanced across participants. Optic fibers 400 μm core in diameter were used to channel the light onto the scalp surface, and 3 mm fiber-optic bundles connected to photomultiplier tubes detected the output light. Fast Fourier transforms were applied to the output current to compute measures of DC (average) intensity, AC (amplitude), and relative phase delay (in picoseconds). The optical data were continuously recorded over each block and sampled at 39.0625 Hz.

**Figure 1:**
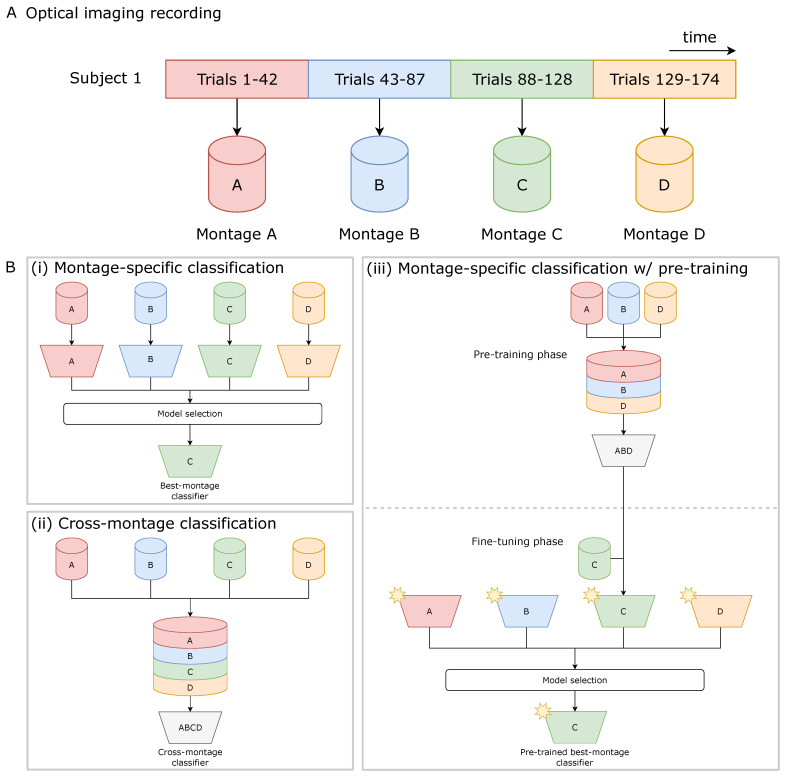
Overview of (**A**) optical imaging recording and (**B**) model training paradigms. Single trials obtained by the same recording montage are color-coded. Training datasets are depicted as cylinders, labeled with the recording montage used for data collection. Convolutional neural network (CNN) models are represented as trapezoids, labeled with the montage data used for model training. In (i) montage-specific classification, a separate model is trained for each montage, and the best-performing model, determined by validation accuracy, is used to evaluate the subject’s held-out test performance. (ii) Cross-montage classification involves training a single model on a concatenated dataset of trials from all montages. (iii) Montage-specific classification with pre-training involves training a model on data from all montages except the montage of interest, followed by fine-tuning the pre-trained model with data from the montage of interest. A star on the trapezoid indicates the use of pre-trained weights.

Sources and detector fibers were secured on the participants’ heads using modified motorcycle helmets. Four recording montages were used to cover the majority of the cortex. Each montage was recorded separately, and the order was counterbalanced across participants. Each montage consisted of 16 detectors coupled with 16 time-multiplexed sources for 256 total channels per montage. Figure 1A provides a streamlined visualization illustrating the use of four montages to record trials for a single participant.

**Figure 2:**
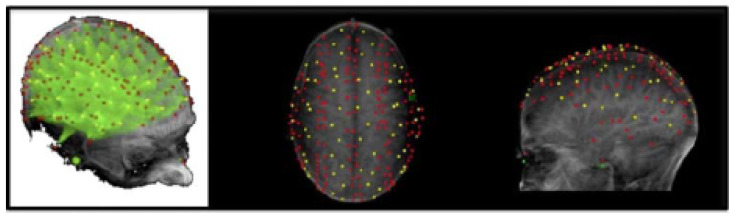
Event-related optical signal (EROS) recording montage with source (red) and detector (yellow) locations. Reprinted from Baniqued et al. [8]. ©2013 MIT. All rights reserved.

The locations of each source and detector in relation to the nasion and fiducial preauricular points were digitized using a Polhemus “3Space” 3-D digitizer (Figure 2). Volumetric T1-weighted (MPRAGE) MR images were acquired for each participant with vitamin E pills positioned on the nasion and preauricular points. The fiducial markers permitted the coregistration of each participant’s digitized optical channels with the corresponding anatomical images. The data were submitted to scalp surface-fitting using a Levenberg–Marquardt algorithm (least-squares fit) and standard Talairach transformation [43].

### 3.4. Fast Optical Signal Pre-Processing

Phase data were corrected for phase wrapping and pulse artifacts [44], adjusted to a mean of zero for each block, and band-pass-filtered between 0.1 and 12 Hz. The data from correct trials were segmented into epochs, time-locked to the onset of the response, using only the manual left/right responses. Channel and baseline correction were applied using a 998 ms period preceding the participant response. Finally, the baseline-corrected data were cropped to 716 ms before and after the response.

**Figure 3:**
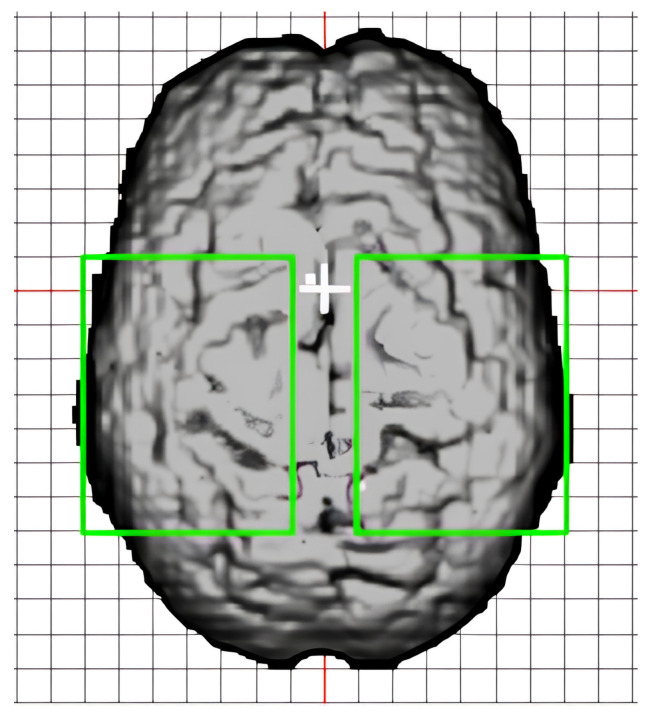
The 6 by 7 cm region-of-interest (ROI) voxel grids (green) for the left and right hemispheres.

Only channels with source–detector distances between 2 and 7 cm, a phase standard deviation of less than 200, and a mean raw AC value over 100 mV were included in the analysis [45]. In-house software “Opt-3d” [46] was used to combine data from channels, creating a 2D axial projection of the 3D voxel space. This was based on the estimation of the diffusion paths for each channel from their source and detector locations and a model of how light diffuses through the head. Single-trial phase and DC data from voxels in a 6 by 7 cm region-of-interest (ROI) centered around the motor cortex of each hemisphere (Figure 3) were used as input to train the deep learning model for left and right-hand responses. This was performed separately for each of the four montage layouts. The spatial dimension was flattened, generating a 2D input matrix of 42 voxels by 56 time points. Statistics (number of trials, viable voxels, and channels per voxel for each subject) for these four montages, indexed by A-D, are summarized in Table 2. Due to the channel inclusion criteria, some subjects have missing data for certain montages. Signals from each voxel were scaled by the maximum absolute value across all voxels and over the time series before input into the model.

### 3.5. Machine Learning Approach

#### 3.5.1. Model Architecture

A modified convolutional neural network (CNN) approach, derived from EEG-based BCIs, was tailored to classify binary motor responses from single-trial EROS data. Table 3 provides a summary of the backbone architecture used in all experiments. This method was selected because of its robust performance across a variety of BCI paradigms, success in limited data regimes, and ability to learn spatial and temporal filters from data [37]. For a detailed visualization of the architecture, readers are referred to Figure 1 in Lawhern et al. [37].

The modified CNN architecture first learns $F_{1}$ convolutional filters of size (1, 20). The filter length was chosen to be half of the data sampling rate (≈40 Hz) to preserve frequency information up to 2 Hz. This convolution operation produces $F_{1}$ feature maps, each representing a band-filtered version of the input signal. For each temporal filter, *D* spatial filters of size (*C*, 1) are trained using depthwise convolution. Here, *C* denotes the flattened dimension of the input space. Batch normalization was employed after each convolutional layer, following the last convolution with an exponential linear unit (ELU) activation and dropout. Consistent with the original implementation, the dropout probability was set to 0.5, the bias parameter was disabled for all convolutional layers, and the maximum norm of the spatial filter weights was constrained to be at most one [37].

Next, the architecture applies a separable convolution, a depthwise convolution of size (1, 20) to summarize 500 ms of activity, followed by $F_{2}$ (1, 1) pointwise convolutions to combine the information obtained from individual spatiotemporal filters. Batch normalization and an ELU activation are applied after the separable convolution. Average pooling with a kernel size of (1, 8) is conducted, along with dropout. The resulting flattened features are then passed through a final linear layer, with the maximum norm of the weights constrained to be at most 0.25. The output of the linear layer is passed through a sigmoid activation function, which maps the output into the range [0,1]. Finally, the final output is compared to a fixed threshold of 0.5 for classification. If the output exceeds the threshold, it is categorized as a right motor response; otherwise, it is classified as a left motor response.

The performance of the proposed CNN model was compared to several baselines, with results summarized in Appendix B. Specifically, the model was trained using the default architecture and hyperparameters proposed for EEG-based BCI by Lawhern et al. [37]. Additionally, performance was compared to the DeepConvNet model proposed by Schirrmeister et al. [26]. The DeepConvNet model, designed as a general-purpose EEG-based BCI architecture for high performance across multiple tasks, was chosen for its potential adaptation to EROS-based BCI tasks.

#### 3.5.2. Model Training

To train each model, an extensive architecture and hyperparameter sweep over $F_{1}$, *D*, $F_{2}$, optimizer, learning rate, weight decay, and validation early stopping metrics was conducted. A total of 50 random hyperparameter configurations were sampled, training each configuration with three random neural network parameter initializations (referred to as iterations). Default hyperparameter configurations, the range of values considered, and the sampling function used to conduct the model architecture and training hyperparameter search are depicted in Appendix A.

Each model was trained for 300 passes over the training data to minimize the binary cross-entropy loss, with early stopping based on the validation set performance. A 20% held-out test set stratified by the labeled response type was used to evaluate the trained models. The remaining 80% of the data was split using 5-fold cross-validation, where 4 of the 5 blocks were used for training and the remaining 1 block was used for validation. All models were trained on an NVIDIA Titan X Pascal GPU, with CUDA 10 and cuDNN v8, in PyTorch [47].

A subject-specific evaluation of the CNN approach was conducted, employing three distinct training paradigms: (i) montage-specific classification, (ii) cross-montage classification, and (iii) montage-specific classification with warm-start initialization of neural network parameters (pre-training). Figure 1B visually describes each of these model training paradigms.

(i) **Montage-specific classification:** A subject-specific model was trained solely on trials obtained from a specific montage and evaluated using the subject’s held-out trials recorded with the same montage. This approach reduced the number of available trials to a maximum of 240 as the data were partitioned into disjoint sets based on the montage configuration used for recording.
(ii) **Cross-montage classification:** The model was trained using trials recorded by all available montages for the subject while ensuring an equal distribution of trials from each montage in the training, validation, and held-out test splits.
(iii) **Montage-specific classification with pre-training:** A warm-start initialization of the CNN parameters was obtained by pre-training models on trials corresponding to all available montages, but excluding the specific montage under evaluation. Identically to the cross-montage case, the training and validation splits were stratified based on montage. The pre-trained model was then fine-tuned using montage-specific data from the previously held-out montage and evaluated the final trained model on the subject’s held-out trials for the corresponding montage.

### 3.6. Evaluation

The reported mean held-out test performance was averaged over the test metrics for the five models obtained via 5-fold cross-validation. These five models were also used to generate the 95% confidence intervals for the test metrics. These models shared the selected hyperparameter configuration yielding the highest validation performance averaged over training iterations. In the analysis of montage-specific classification methods (i and iii), classification accuracy and the area under the Receiver Operating Characteristic curve (AUROC) on the held-out test set were presented for the specific montage model that achieves the highest validation accuracy. The selection of the “best montage“ for each subject precedes the final evaluation of the montage-specific model on the independently sampled held-out test set. To illustrate, suppose a subject had trained models for montages A, B, C, and D, and, after assessing various hyperparameter settings, the model trained on trials from montage C exhibited the highest average validation accuracy across training iterations. In this case, the held-out test metrics associated with montage C would be reported as the subject’s “best montage” performance. This approach allows for the aggregation of a subject’s performance across montage-specific metrics, as good signal quality is anticipated from only a subset of available montage configurations.

Within-subject classification results are reported for several experimental conditions, in which the following variables are varied:

- **Input signal:** Phase delay and intensity data were recorded for each participant. The following inputs were explored to train the proposed method: (i) only phase signals, (ii) only intensity signals, and (iii) both phase and intensity signals, recorded simultaneously.
- **Frequency band:** This study identifies informative frequency ranges for event-related activity occurring at specific frequencies (see Table 4 for frequency ranges of interest). Additionally, it assesses the CNN’s capability to automatically learn temporal filters compared to the conventional practice of manually selecting frequency bands, which has been commonly employed in the existing literature. The narrow frequency band analysis focuses on intensity in dual-input experiments using both phase and intensity input signals, given that intensity is particularly susceptible to the masking of discriminative information by low-frequency noise contamination.
- **Training paradigm:** Three distinct neural network training paradigms were used, and each was evaluated separately on the 0.1–12 Hz filtered phase input and intensity input: (i) montage-specific, (ii) cross-montage, and (iii) montage-specific with pre-training. In the case of dual-input experiments, separate spatiotemporal CNN architectures were initialized for each input type. These parallel models were trained jointly, and the flattened outputs from each model were combined and passed through a final dense layer for prediction.

Finally, this study investigated the correlation between montage recording quality metrics and classification accuracy within the montage-specific classification setting. Two signal quality measures for each montage were considered:

- **Viable voxel count:** The quality of montage placement was quantified by determining the number of voxels that contain viable channels mapping to that voxel. This measure provides insight into how well the recording montage covers the region of interest, resulting in signal recording focused around the discriminative region.
- **Channels per voxel:** The average number of channels mapping to each voxel, averaged over all viable voxels within a montage, was computed. This measure evaluates the robustness of the recorded signal to noise disturbances, as voxels with a higher number of averaged channels tend to have a higher signal-to-noise ratio.

These signal quality measures are summarized for each subject and montage in Table 2.

### 3.7. Feature Explainability

The recent development of methods for deep neural network feature explainability has enabled the interpretation and visualization of features learned by “black box” models, allowing practitioners to take a step toward explaining complex classification decision rules [48,49,50,51,52,53,54]. Specifically, feature attribution methods can highlight the most influential features in a model’s prediction [49,50,53,54]. This study focuses on identifying specific spatial regions and temporal intervals contributing to a single-trial classification decision by the model, where a positive feature relevance indicates information supporting the decision and vice versa. Utilizing DeepLIFT (with the Rescale rule), a gradient-based feature attribution method, the feature relevance at the first convolutional layer is computed, assigning relevance values to each voxel and time step of the input signal [50].

## 4. Results

### 4.1. Subject-Specific Classification Performance

**Figure 4:**
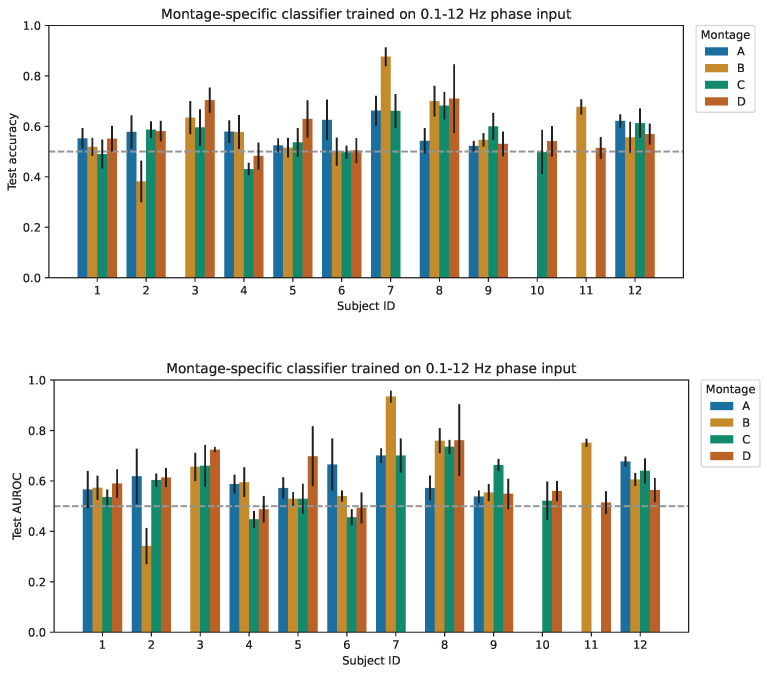
Held-out test accuracy and AUROC for the proposed CNN model trained on 0.1–12 Hz band-filtered phase data from a given subject and montage. Subjects 7, 10, and 11 have missing data from one or more montages. The error bars depict the 95% confidence interval about the mean, generated using reported test metrics from 5-fold cross-validation. The gray dashed line represents the random chance classifier (50.0% accuracy and AUROC of 0.500 for a binary classification task).

Table 4 shows subject-specific classification results aggregated over all participants. The spatiotemporal CNN trained on 0.1–12 Hz filtered phase input and trained by selecting the best-performing montage-specific classifier for each subject achieved a test accuracy of 62.8% (95% CI: [0.574, 0.682]) and AUROC of 0.674 (95% CI: [0.615, 0.733]) averaged across 12 participants. This experimental setup demonstrates the highest classification performance, with a significant majority of the subjects achieving an accuracy above the chance level (p<0.05). Notably, all participants achieved statistically significant AUROC values (p<0.05). Refer to Figure 4 for detailed visualizations of individual subject and montage-specific performance metrics.

This study demonstrates that the proposed CNN method, utilizing optimized architecture parameters (specifically, F1, *D*, F2 for the number of convolutional filters) and refined hyperparameter tuning, achieves comparable average cross-subject classification performance and a greater proportion of subjects performing above chance levels compared to existing CNN baseline methods (Table 5). Comprehensive details regarding the implementation of these baseline methods are available in Appendix B. Throughout the subsequent analysis, emphasis is placed on classification accuracy results, given that the reported held-out AUROC values follow a similar ranking across approaches.

For the statistical testing, we employed a Type-II Analysis of Variance (ANOVA) to model the classification test accuracy as the response variable, with experimental setup and subject number as factors. The analysis identified significant interaction effects between these factors and distinct group mean differences. Post hoc pairwise Tukey honestly significant difference (HSD) tests further examined the differences in group means. In contrast to the best-montage model, both cross-montage and pre-trained models exhibited lower average test accuracies for the phase signal, though only the cross-montage model differences were statistically significant (p<0.05).

The models trained exclusively on intensity data exhibited the lowest average classification accuracy, failing to exceed chance levels across all experimental configurations. However, individual subject analysis revealed that six subjects achieved above 50% accuracy in at least one training paradigm using intensity inputs. Moreover, these subjects also achieved a test accuracy above 50% for some combination of phase and training paradigms. This finding raises the question of whether different, yet complementary, features may be extracted from the different input types. Motivated by these findings, we conducted dual-input experiments to explore improving subject performance by combining phase and intensity data modalities. The implications of these results are discussed in more depth in Section 5.

Furthermore, no significant benefit from utilizing a narrow frequency band for the phase input was observed, as fewer than half of the subjects performed better than random chance (p>0.05). No significant difference in performance from incorporating both phase and intensity input into a dual-input model was observed, regardless of the frequency band used for the intensity input (p>0.05). This analysis assessed the presence/absence of discriminative information in event-related activity at specific frequencies, rather than the power variation across frequencies as in a time–frequency analysis. Consequently, it is expected that restricting information to a subset of frequencies within the broadband (0.1–12 Hz) range would not enhance performance, as much of the discriminative signal may be diminished during narrow band filtering.

### 4.2. Correlation between Data Quality and Montage-Specific Performance

The highest-performing experimental setup described in the previous subsection (0.1–12 Hz filtered phase input trained with the montage-specific paradigm) provided a baseline for the proposed approach’s performance with a fixed data size and non-variable sensor configuration. The relationship between data quality measures and classification accuracy was statistically analyzed using a Type-II ANOVA and is summarized in Table 6. The classification test accuracy was treated as the dependent variable and the data quality measure (number of viable voxels and average number of channels per viable voxel) and subject number were treated as factors. This analysis revealed that the average number of channels per viable voxel had a significant positive effect on classification accuracy for a given subject and montage (p<0.05).

When hemisphere-specific data quality measures were considered in the ANOVA, the number of viable voxels in the right hemisphere had a significant positive effect on the classification accuracy (p<0.05), but the number of viable voxels in the left hemisphere did not exhibit a significant effect (p>0.05). These preliminary findings provide insights into the relationship between signal quality, sensor placement, and classification performance. Specifically, the fact that only right hemisphere data displayed sensitivity to the number of viable voxels may suggest that this hemisphere’s data were more critical for classification. This could be because all the participants were right-handed. In right-handed subjects, the right hemisphere is activated only during contralateral movements, whereas the left hemisphere becomes active during both contra and ipsilateral movements, leading to less hand-specific specificity in the left hemisphere [55].

### 4.3. Correlation between Data Quality and Cross-Montage Performance

This study also investigated the correlation between the classification performance of montage-specific models trained on 0.1–12 Hz filtered phase input and the classification performance of multi-montage training paradigms (cross-montage and pre-trained models). Specifically, the effects of the following montage-specific measures on multi-montage classification accuracy were investigated: the number of available training montages, the number of montages with montage-specific classification accuracy above 50%, and the proportion of montages with montage-specific classification accuracy above 50%.

A Type-I ANOVA was used for this analysis as no significant interaction effects between the subject identifier and montage-specific measures were anticipated. The results revealed that the number of available montages and the number of montages with above-chance accuracy for a given subject significantly contributed to the classification accuracy of the cross-montage training paradigm (p<0.05). However, the proportion of montages with above-chance accuracy does not yield a significant effect for this paradigm (p>0.05). In contrast, for the pre-training paradigm, both the number and proportion of montages with above-chance accuracy positively affected the classification accuracy (p<0.05). These results provide valuable insights into the effectiveness of different training paradigms for utilizing multi-montage data, and their implications are discussed in more detail in Section 5.

### 4.4. Neural Network Feature Relevance

**Figure 5:**
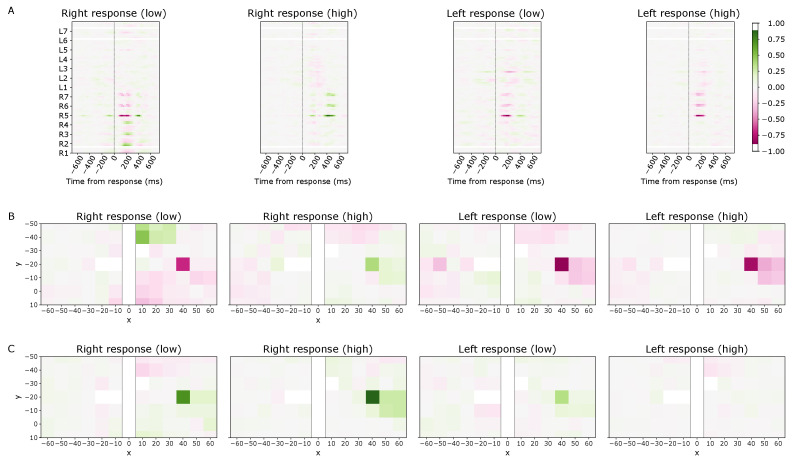
Trained neural network DeepLIFT feature attributions for various single-trial response types and prediction confidence levels (shown in parentheses). The proposed convolutional neural network (CNN) model was trained on 0.1–12 Hz band-filtered phase data. Green indicates positive relevance values and red indicates negative relevance, though magnitude is the primary informative descriptor. (**A**) Input relevance as a function of space (y-axis) and time (x-axis). Spatial indices represent top-down rows of the region-of-interest (ROI) for each L/R hemisphere. (**B**) ROI voxel relevance at 179 ms after the response and (**C**) ROI voxel relevance at 410 ms after the response. The x and y axes represent Talairach coordinates (mm).

Neural network feature relevance values were computed for the best-performing (0.1–12 Hz phase, montage-specific) model trained on Subject 7’s data recorded from montage B. Selecting a model with high classification accuracy enables the analysis of high-confidence (prediction <0.20 or >0.80) and low-confidence (prediction between 0.20 and 0.80) single-trial model predictions. To visualize general trends, trials were grouped by response type and confidence level and then averaged to yield the feature relevance heatmaps in Figure 5.

Analyzing the contributions of specific time points in the input signal to the model prediction over time reveals larger magnitude contributions after the time of the response, specifically centered around 179 ms and 410 ms after the response for both right and left-handed responses. Furthermore, there is a greater activation magnitude in the right hemisphere than in the left, supporting the hypothesis that the signal from the right hemisphere is more discriminative. For correctly classified and high-confidence right-handed responses, there is a strong positive (green) contribution by the signal recorded 410 ms after the response. For correctly classified and high-confidence left-handed responses, there is a strong negative (red) contribution by the signal recorded 179 ms after the response. In the case of low-confidence predictions for both response types, both of the aforementioned contributions are visible and these trials have more noisy feature attributions in general, thus leading to less discriminability between the left and right response types.

To identify the contributions of spatial locations within the ROI, this study visualized feature importance values at the time points with the largest magnitude of contribution: 179 and 410 ms after the response. Again, the contrast between activation magnitude between the hemispheres is apparent. Additionally, the polarity of activation at location (40, −20) for right-handed versus left-handed responses is switched at 179 ms after the response, indicating a high discriminative signal. This voxel corresponds to the hand region of the motor cortex, which reflects the hand-related motor movement and shows the highest magnitude of activation for high-confidence model predictions. For lower-confidence predictions, the contributions are less specific and attribute spatial importance to larger brain regions.

## 5. Discussion

This study explored the potential of using a convolutional neural network (CNN) architecture that learns temporal and spatial filters to classify EROS in a single-trial setting. It considered several variations of the analytical setup, varying the input signal (phase delay and intensity), the temporal filtering applied during pre-processing, and the neural network training paradigm (single-montage, cross-montage, pre-trained single-montage). To date, this study represents the first attempt to apply deep learning techniques to the single-trial classification of EROS signals. The findings demonstrate that a CNN model trained on phase data can effectively learn discriminative features for motor response classification, achieving an average accuracy of 62.8% and AUROC of 0.674 across 12 participants. Additionally, the relationship between sensor coverage of the discriminative region of interest and the performance of montage-specific models was investigated. The analysis reveals a positive correlation between the average number of channels per voxel recorded with a montage and the classification accuracy achieved by that montage, indicating that classification accuracy critically depends on data quality. Furthermore, the consistency of performance across the different montages recorded for each subject indicates the potential effectiveness of multi-montage training paradigms in achieving high classification performance. Overall, the present study contributes to a better understanding of the data conditions required to successfully apply deep learning approaches to BCI tasks, specifically in the context of EROS signals.

Although the classification accuracy did not meet the 70% threshold considered essential for effective BCI communication [56], these findings are consistent with prior work achieving 63% accuracy in single-trial classification EROS for retinotopy [41] and 63% accuracy in an online visual oddball task utilizing an average of 15 trials [7]. Furthermore, this study shows that the proposed method, incorporating architecture search and hyperparameter tuning, maintains consistent performance comparable to established CNN approaches. Moreover, the proposed method yields the highest proportion of subjects achieving above-chance classification accuracy and AUROC, underscoring the importance of subject-specific hyperparameter optimization.

The montage-specific paradigm trained on a relative broadband 0.1–12 Hz filtering of phase data achieved the highest performance across participants. This finding is consistent with prior research suggesting the superior classification performance of phase compared to intensity data for classifying Fast Optical Signals (FOS) [57,58]. These earlier studies proposed that external noise sources may affect intensity measurements, potentially reducing their discriminative power. However, it is important to note that some previous investigations have reported the opposite trend, with intensity data outperforming phase data [59,60]. More recently, a cross-subject study employing support vector machines (SVMs) for classifying EROS signals found intensity data to be superior to phase data [41]. The authors of that study hypothesized that the lower performance of phase data could be attributed to the challenge of aligning sensor locations to the brain across participants. Phase delay signals have a higher spatial resolution than intensity signals, making them more sensitive to spatial shifts in channel locations [61]. This inter-subject variability in channel alignment could impact the classification performance when using subject-averaging approaches.

The present study bypasses this limitation by developing subject-specific classifiers, eliminating the potential influence of inter-subject variability on sensor alignment. Moreover, the superiority of the single-montage training paradigm over multi-montage approaches in the results further supports the notion that phase data are sensitive to spatial shifts across montages since common features are difficult to learn across variable montages. However, the limited number of available trials for each subject–montage combination likely impaired the training of single-montage classifiers, contributing to the low average accuracy across subjects [56]. To better evaluate the single-subject classification performance of EROS, experiments with a greater number of trials and a single recording montage should be conducted as larger datasets generally enhance the performance of machine learning frameworks. In the absence of additional data availability, data augmentation strategies may be employed to simulate a larger dataset [26].

Alternatively, future studies could consider using subject-specific structural images, such as anatomical magnetic resonance images, to accurately align optical channels with distinct brain anatomy before voxel-space reconstruction and mitigate this limitation. This procedure holds the potential to reduce the impact of spatial shifts and enhance the classification performance of phase data when employing multi-montage training paradigms.

No performance gains were observed when pre-processing the default 0.1–12 Hz input signal using band-pass filtering. This finding indicates that domain expertise in selecting narrow frequency bands of interest is unnecessary for achieving good classification performance with the CNN approach, which automatically extracts temporal features. The added restriction of fixed frequency ranges may impede the learning of temporal features that span multiple frequency ranges (4–7, 8–13, and 13–20 Hz) considered in this study. This limitation is particularly plausible given the variability of inter-subject signals, as frequency-based features are not expected to generalize across subjects. The broad frequency range of the 0.1–12 Hz input allows for the model to flexibly learn the most discriminative frequency information for each subject.

As stated in Section 4.1, the investigation reveals that a subset of subjects achieving a phase-based classification accuracy exceeding 50% also demonstrate accuracy above 50% with intensity. This observation prompted whether dual-modality analyses might be more effective than single-modality paradigms for classification purposes. In these analyses, separate CNNs for each modality (phase and intensity) were trained and their features were concatenated at the final classification layer. However, no significant improvement or deterioration in classification accuracy was found when both phase and intensity inputs were employed, compared to using phase input alone, suggesting that the two modalities may provide redundant information. The effects of this phenomenon might have been confounded by the limited dataset size. Consequently, it is imperative to conduct future studies utilizing more abundant data to fully assess the potential of dual-modality feature extraction.

This study investigated the impact of data quality on the classification performance of the proposed method. Quantitatively, it identified a positive statistical effect between the average number of channels per viable voxel in a montage on the held-out classification accuracy of that montage. Specifically, significant effects were observed in the number of viable voxels in the right hemisphere, whereas no significant effects were found in the left hemisphere. It is hypothesized that the right hemisphere ROI contains a more discriminative signal because, for right-handed participants, the left hemisphere’s activation is bi-lateral (activated for both right and left motor responses), whereas the right hemisphere is expected to be activated mainly with left motor responses. These results highlight that the data quality in the discriminative region of interest is important in predicting classification performance.

Similarly, the study aimed to identify a good predictor of multi-montage performance, specifically for the cross-montage and pre-trained montage-specific paradigms. The findings indicated a significant positive impact on classification accuracy within the cross-montage training paradigm when considering two factors: the number of available montages and the number of montage-specific models achieving above 50% accuracy for a given subject. The observed correlation between the number of available montages and classification performance can be attributed to the enlarged training dataset resulting from the concatenation of all available montage data. Additionally, the correlation between the number of montages with above 50% accuracy and cross-montage performance suggests that an increased number of properly aligned montages with discriminative regions of interest contributes to superior performance. Furthermore, it is possible that subjects with high performance exhibit better alignment among montages, leading to similar signals and reduced noise in the concatenated dataset.

The present study also demonstrates that both the number and proportion of available montages exhibiting classification accuracy above 50% have a significant positive effect on the accuracy achieved through the pre-trained single-montage training paradigm. Assessing the proportion of high-performing montages provides a less restrictive measure of montage data quality, as it accounts for instances where certain subjects lack montage data but can still benefit from the advantageous inductive biases derived from the pre-training step, thereby facilitating downstream task-specific fine-tuning. In contrast to the cross-montage approach, where the alignment of all montages is crucial, the alignment of all montages is less essential for this paradigm since the neural network weights are fine-tuned specifically for a target montage. Additionally, the number of available montages has a limited impact on performance within this paradigm due to the potential for negative inductive biases to arise from pre-training on dissimilar montage data, thereby impairing performance. Therefore, it is more advantageous to include a few montages with a discriminative signal rather than introducing additional noisy montages into the training paradigm. It is postulated that the performance could be further enhanced by pre-selecting high-performing montages for inclusion in the concatenated dataset during the training (cross-montage) or pre-training (pre-trained montage-specific) phase. However, this investigation is deferred to future work.

It must be noted that accurate alignment of the optical channels using subject-specific anatomical features has the potential to render the distinction between single-montage and multi-montage approaches obsolete, as it enables the representation of all montage signals within the same voxel space. Future work may also investigate the alignment of multiple subject data to develop a cross-subject classifier.

Finally, the visualization of feature relevance attributing the neural network’s prediction to specific temporal intervals and spatial regions of the input provides valuable insights for the broader scientific community. The findings of these analyses support that the most discriminative signals originate from the motor cortex, aligning with expectations for such a motor response task. Furthermore, these interpretations help elucidate the importance of collecting high-quality data from discriminative regions of interest in future investigations.

## 6. Conclusions

This study explores the use of a convolutional neural network (CNN) architecture to classify event-related optical signals (EROS) in a single-trial setting. The proposed method was evaluated on a data set from a previous study in which the participants performed a motor response task [8]. The implemented method utilizes a series of two-dimensional convolutions to learn temporal and spatial filters from the EROS data. This study demonstrates that the CNN model trained on phase data, utilizing a montage-specific approach, achieves an average accuracy of 62.8% for motor response classification, surpassing the models trained on intensity data. This suggests some discriminative power of phase data for EROS classification tasks. Furthermore, a positive correlation between the sensor coverage of the discriminative region and the classification accuracy was observed, indicating the importance of comprehensive coverage in data collection methodologies for future EROS studies.

Among various subject-specific model training paradigms, the montage-specific training paradigm yielded the highest performance. Data quality, measured by the number of available voxels and the average number of channels per voxel, significantly improved classification performance in single-montage training. In the cross-montage training paradigm, the number of available montages and the number of montages achieving classification accuracy above random chance positively impacted accuracy. The number and proportion of montages achieving above-chance accuracy in the pre-training paradigm had a significant positive effect. A post-hoc analysis of learned feature relevance identified discriminative time intervals and spatial locations of the input signal, highlighting the hand motor cortex in the right hemisphere as a significant contributor to the model’s predictions. Finally, the proposed method’s classification efficacy is comparable with existing CNN-based brain–computer interface (BCI) research using electroencephalography (EEG) and is consistent with both the multi-trial and single-trial EROS classification literature.

Future investigations should further explore multi-montage model-training paradigms and incorporate subject-specific or inter-subject alignment of optical channels to enhance classification performance and increase the dataset size. Furthermore, investigating data augmentation strategies to simulate a larger effective dataset size is crucial for enhancing the efficiency and robustness of training deep learning models.

Although achieving higher classification accuracy is desirable, this study highlights the potential of using deep learning approaches to automate feature extraction for EROS signals, which holds promise for various BCI applications.

## Figures and Tables

**Table 1:** Summary of related works using machine learning algorithms for electroencephalography (EEG)- and event-related optical signal (EROS)-based brain–computer interface (BCI) applications. Abbreviations are summarized at the end of the main text.

Author	BCI Application	ML Algorithm(s)	Data	Limitation
[17]	Motor imagery	LDA, RFD, SVM	EEG	Spectral density features limit applicability in online settings
[18]	Motor imagery, finger movement	LDA, QDA, KFD, SVM, MLP, LVQ, k-NN, DT	EEG	Reliance on task-specific feature extraction
[19]	Motor imagery	LDA, NDA, SVM	EEG	Reliance on task-specific feature extraction and selection
[22]	Motor imagery	CNN, SAE	EEG	Domain expertise required for frequency- band pre-processing, large trainable parameter count
[23]	Motor imagery	CNN	EEG	Feature extraction independently considered from classification algorithm, separate spatial filter for each pairwise combination of classes
[24]	Motor imagery	CNN	EEG	Discretized frequency subbands limit method’s capacity to identify trends across larger frequency ranges
[25]	Motor imagery	CNN	EEG	Large trainable parameter count
[26]	Motor imagery, hand and foot movement	CNN	EEG	Minimal performance gains over baselines in limited data regime
[37]	Various ERP- and oscillatory- based tasks	CNN	EEG	Subject-specific hyperparameter and architecture selection has yet to be explored
[5]	Object recognition	ICA	EROS	Event-related averaging across a large number of trials
[6]	Go-NoGo	ICA	EROS, EEG	Event-related averaging across a large number of trials
[7]	Visual oddball classification	LDA, SVM	EROS	Event-related averaging across a large number of trials, domain expertise required for feature extraction
[41]	Retinotopy classification	SVM	EROS	Domain expertise required for frequency-based feature extraction

**Table 2:** Left: Total number of correct trials recorded for each subject and montage. Center: The number of voxels (out of 98) containing a non-zero number of channels. Right: The number of channels per voxel averaged over all non-zero voxels. Entries corresponding to subjects without specific montage recordings are left blank.

Subject ID	Total Number of Trials				Viable Voxel Count				Channels Per Voxel			
	**A**	**B**	**C**	**D**	**A**	**B**	**C**	**D**	**A**	**B**	**C**	**D**
1	208	219	196	222	78	77	79	70	3.9	4.5	5.3	3.4
2	225	218	222	211	83	80	75	72	4.0	4.6	4.4	4.6
3		229	229	228		84	65	60		3.7	2.3	1.6
4	233	219	194	226	80	77	70	75	4.3	3.9	3.9	4.0
5	199	190	204	200	81	79	79	75	3.6	4.1	4.2	3.3
6	228	229	223	226	84	84	74	72	4.7	4.4	4.6	5.4
7	222	210	226		81	81	78		4.3	4.6	4.6	
8	208	210	220	196	78	83	76	75	4.8	5.0	5.0	5.0
9	177	221	170	212	83	76	75	75	3.9	3.7	4.4	4.6
10			225	220			72	74			4.2	4.4
11		220		210		84		74		3.8		5.1
12	229	228	228	228	81	75	77	73	4.5	3.7	4.1	4.1

**Table 3:** Depthwise separable convolutional neural network (CNN) architecture, where *C* is the number of input channels, *T* is the number of time points, $F_{1}$ is the number of temporal filters, *D* is the number of spatial filters per temporal filter, and $F_{2}$ is the number of pointwise filters.

Layer	# Filters	Size	# Params	Output	Activation	Options
Input				(*C*, *T*)		
Reshape				(1, *C*, *T*)		
Conv2D	$F_{1}$	(1, 20)	20 ∗ $F_{1}$	($F_{1}$, *C*, *T*)	Linear	mode = same
BatchNorm			2 ∗ $F_{1}$	($F_{1}$, *C*, *T*)		
DepthwiseConv2D	*D* ∗ $F_{1}$	(*C*, 1)	*C* ∗ *D* ∗ $F_{1}$	(*D* ∗ $F_{1}$, 1, *T*)	Linear	mode = valid, depth = *D*,max norm = 1
BatchNorm			2 ∗ *D* ∗ $F_{1}$	(*D* ∗ $F_{1}$, 1, *T*)		
Activation				(*D* ∗ $F_{1}$, 1, *T*)	ELU	
Dropout				(*D* ∗ $F_{1}$, 1, *T*)		p=0.5
SeparableConv2D	$F_{2}$	(1, 20)	20 ∗ *D* ∗ $F_{1}$ + $F_{2}$ ∗ (*D* ∗ $F_{1}$)	($F_{2}$, 1, *T*)	Linear	mode = same
BatchNorm			2 ∗ $F_{2}$	($F_{2}$, 1, *T*)		
Activation				($F_{2}$, 1, *T*)	ELU	
AveragePool2D		(1, 8)		($F_{2}$, 1, *T* // 8)		
Dropout				($F_{2}$, 1, *T* // 8)		p=0.5
Flatten				($F_{2}$ ∗ (*T* // 8))		
Dense	$F_{2}$ ∗ (*T* // 8)			1	Sigmoid	max norm = 0.25

**Table 4:** Average held-out test performance metrics and 95% confidence interval across subjects (N = 12) for each input signal, frequency range, and training approach combination. The highest overall test accuracy and area under the Receiver Operating Characteristic curve (AUROC) were obtained by selecting the best-performing montage-specific classifier for each subject based on the validation accuracy, and the held-out test metrics for this experimental setup are shown in bold. The column to the right of each metric column depicts the proportion of subjects that achieve classification performance above random chance (Accuracy: 50.0%, AUROC: 0.500).

Input Signal	Freq. (Hz)	Approach	Accuracy	>50%	AUROC	>0.5
Phase	0.1–12	Best Montage	**0.628 ± 0.054**	**10/12**	**0.674 ± 0.059**	**12/12**
Phase	0.1–12	Cross-Montage	0.559 ± 0.042	8/12	0.580 ± 0.054	8/12
Phase	0.1–12	Pre-Train Best Montage	0.597 ± 0.061	7/12	0.628 ± 0.074	8/12
Phase	4–7	Best Montage	0.519 ± 0.031	5/12	0.515 ± 0.037	3/12
Phase	8–13	Best Montage	0.506 ± 0.024	1/12	0.514 ± 0.031	1/12
Phase	13–20	Best Montage	0.477 ± 0.028	1/12	0.472 ± 0.040	0/12
Intensity	0.1–12	Best Montage	0.490 ± 0.025	1/12	0.484 ± 0.036	2/12
Intensity	0.1–12	Cross-Montage	0.502 ± 0.013	1/12	0.506 ± 0.019	2/12
Intensity	0.1–12	Pre-Train Best Montage	0.501 ± 0.036	4/12	0.501 ± 0.041	5/12
Intensity	4–7	Best Montage	0.507 ± 0.022	3/12	0.4950. ± 027	3/12
Intensity	8–13	Best Montage	0.522 ± 0.019	2/12	0.530 ± 0.029	3/12
Intensity	13–20	Best Montage	0.496 ± 0.025	2/12	0.496 ± 0.031	2/12
Phase + Intensity	0.1–12 + 0.1–12	Dual-Input Best Montage	0.580 ± 0.051	7/12	0.601 ± 0.067	7/12
Phase + Intensity	0.1–12 + 8–13	Dual-Input Best Montage	0.592 ± 0.051	7/12	0.608 ± 0.070	8/12
Phase + Intensity	0.1–12 + 13–20	Dual-Input Best Montage	0.586 ± 0.057	6/12	0.625 ± 0.069	7/12

**Table 5:** Average held-out test performance metrics and 95% confidence interval across subjects (N = 12) for the proposed deep learning method, DeepConvNet [26] baseline, and the default EEGNet [37] architectures. All experiments were conducted with 0.1–12 Hz band-filtered phase input, and the test performance was averaged over the best subject-specific montage configuration. The highest overall test accuracy and area under the Receiver Operating Characteristic curve (AUROC) were obtained by the proposed method, and the held-out test metrics for this architecture are shown in bold. The column to the right of each metric column depicts the proportion of subjects that achieved classification performance above random chance (Accuracy: 50.0%, AUROC: 0.500).

Input Signal	Freq. (Hz)	Approach	Accuracy	>50%	AUROC	>0.5
Phase	0.1–12	Proposed CNN	**0.628 ± 0.054**	**10/12**	**0.674 ± 0.059**	**12/12**
Phase	0.1–12	DeepConvNet	0.609 ± 0.094	7/12	0.635 ± 0.115	8/12
Phase	0.1–12	EEGNet-8,2	0.604 ± 0.104	8/12	0.650 ± 0.118	9/12
Phase	0.1–12	EEGNet-4,2	0.612 ± 0.093	7/12	0.643 ± 0.112	8/12

**Table 6:** Summary of Analysis of Variance (ANOVA) investigating the correlation between data quality and classification performance for the proposed convolutional neural network (CNN) model trained on 0.1–12 Hz phase input. The dependent variable was held-out test classification accuracy. A Type-II ANOVA was used for the montage-specific experiments, and a Type-I ANOVA was used for the cross-montage experiments.

Training Paradigm	Data Quality Measure	*p*-Value
Montage-specific	# viable voxels in L and R hemi.	0.272
Montage-specific	# viable voxels in R hemi.	0.030
Montage-specific	# viable voxels in L hemi.	0.366
Montage-specific	avg. # channels / viable voxel in L and R hemi.	0.048
Cross-montage	# training montages	<0.001
Cross-montage	# montages w/ acc. >50%	<0.023
Cross-montage	prop. montages w/ acc. >50%	0.920
Pre-train	# training montages	0.514
Pre-train	# montages w/ acc. >50%	<0.001
Pre-train	prop. montages w/ acc. >50%	<0.001

**Figure A1:**
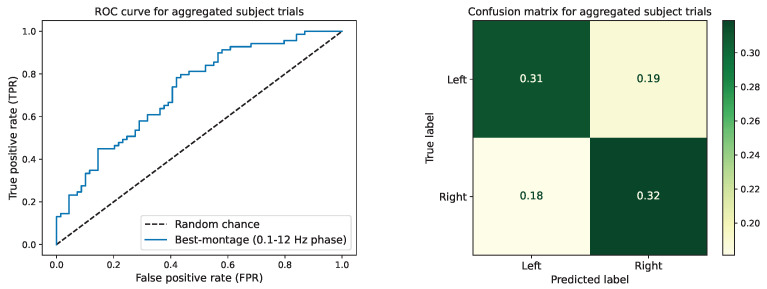
Receiver Operating Characteristic (ROC) curve and confusion matrix for predictions on the concatenated held-out test set for all subjects. Predictions were generated by the best-performing montage convolutional neural network (CNN) trained on 0.1–12 Hz phase input and aggregated across all montage-specific subject trials.

**Figure A2:**
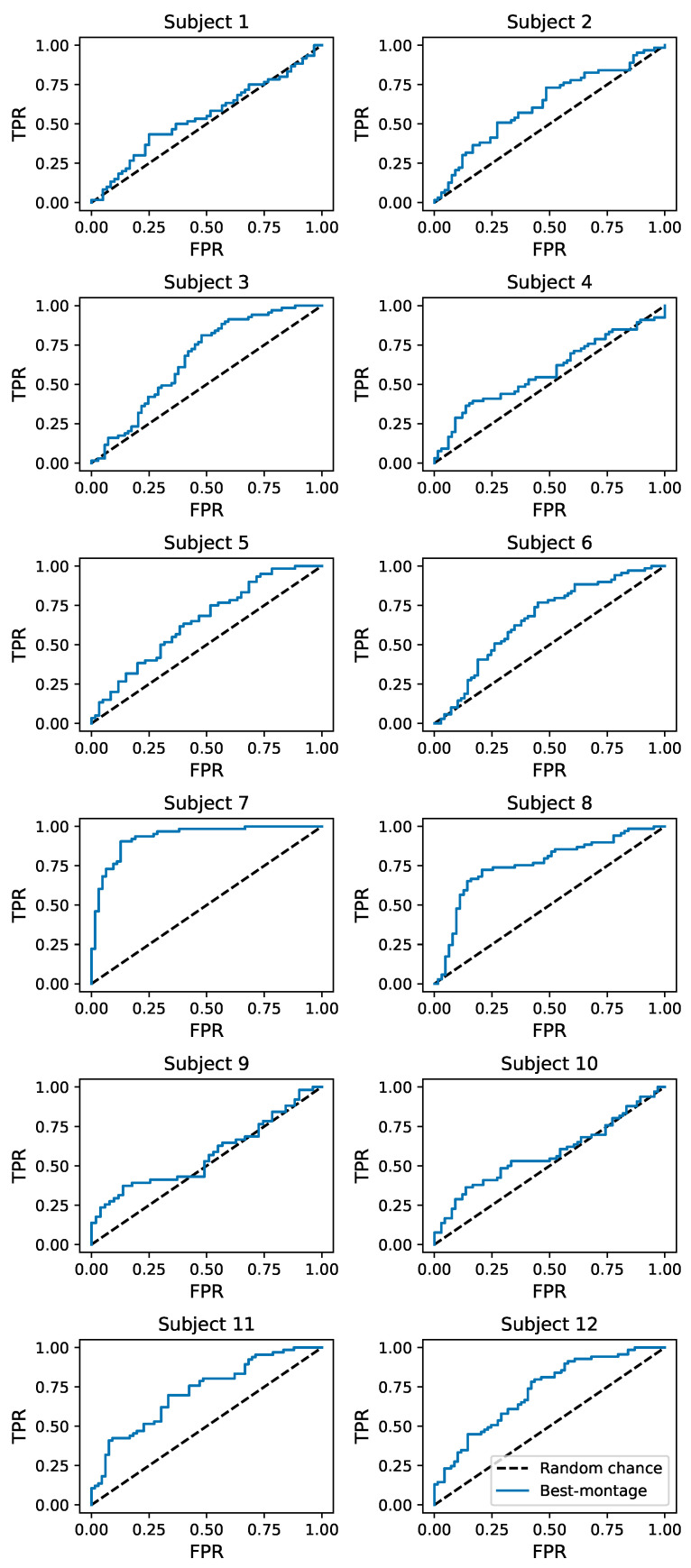
Receiver Operating Characteristic (ROC) curves for individual subjects, with the false positive rate (FPR) on the x-axis and true positive rate (TPR) on the y-axis. Predictions were obtained by evaluating each subject’s best-performing montage convolutional neural network (CNN), trained on 0.1–12 Hz phase input, on the subject’s held-out test trials. Predictions were aggregated across all model training iterations.

**Figure A3:**
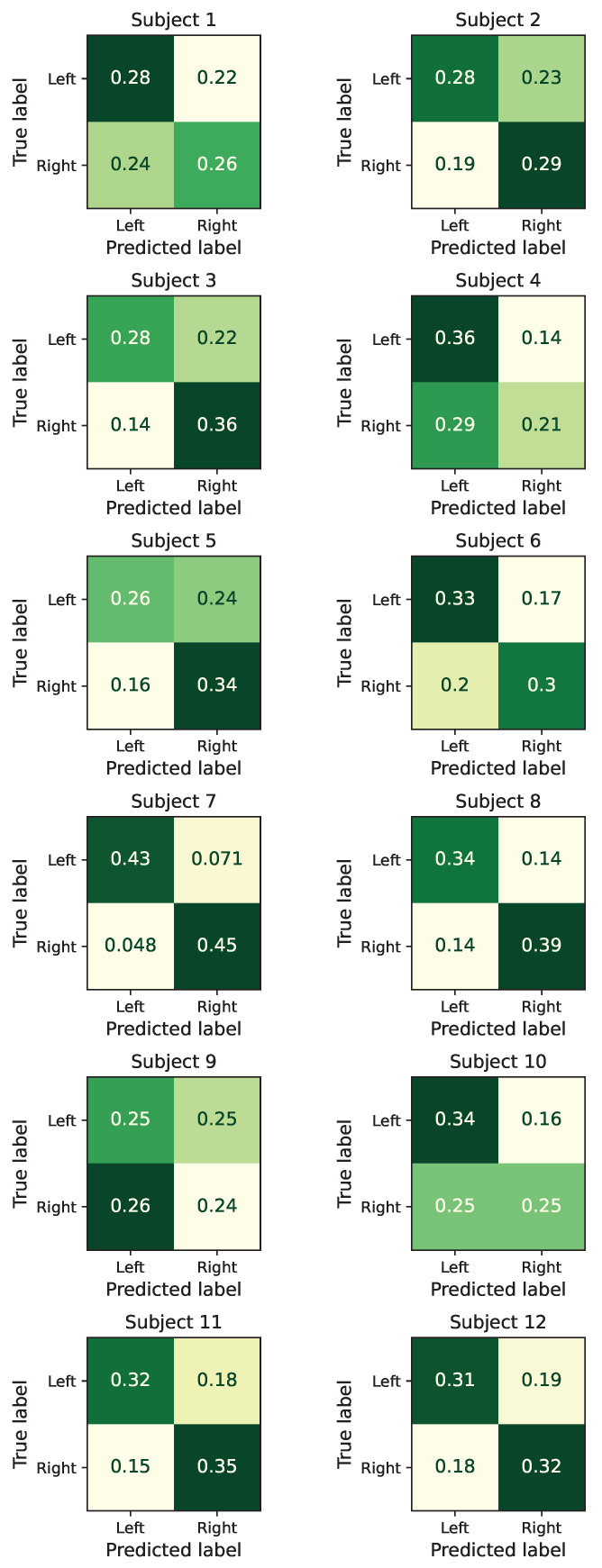
Confusion matrices for individual subjects. Predictions were obtained by evaluating each subject’s best-performing montage convolutional neural network (CNN), trained on 0.1–12 Hz phase input, on the subject’s held-out test trials. Predictions were aggregated across all model training iterations.

## Data Availability

The data presented in this study are available on request from the corresponding author due to privacy restrictions.

## Abbreviations

The following abbreviations are used in this manuscript:

AC	alternating current light intensity
ANOVA	Analysis of Variance
AUROC	area under the curve of the Receiver Operating Characteristic
BCI	brain–computer interface
CI	confidence interval
CNN	convolutional neural network
CSP	common spatial patterns
CW	continuous-wave
DC	direct current light intensity
DT	decision tree
EEG	electroencephalography
EHR	electronic health records
ELU	exponential linear unit
EROS	event-related optical signals
FD	frequency-domain
FBCSP	filter bank common spatial patterns
fNIRS	functional near-infrared spectroscopy
FOS	fast optical signals
FPR	false positive rate
HSD	honestly significant difference
ICA	independent component analysis
KDA	kernel Fisher discriminant analysis
k-NN	k-nearest neighbors
LDA	linear discriminant analysis
LVQ	learning vector quantization
MI	motor imagery
MLP	multi-layer perceptron
MPRAGE	magnetization-prepared rapid gradient-echo
MR	magnetic resonance
NDA	nonlinear discriminant analysis
QDA	quadratic discriminant analysis
RFD	regularized Fischer discriminant
ROC	Receiver Operating Characteristic
ROI	region-of-interest
SAE	stacked autoencoder
SGD	stochastic gradient descent
SNR	signal-to-noise ratio
STFT	short-time Fourier transform
SVM	support vector machine
TPR	true positive rate

## Appendix A. Hyperparameter Tuning

To optimize the neural network model hyperparameters, an architecture search was conducted across varying numbers of temporal ($F_{1}$), spatial (*D*), and pointwise ($F_{2}$) filters alongside a hyperparameter sweep over the choice of the optimizer, learning rate, weight decay, and early stopping metric. The ranges of values considered and the sampling function used for both model architecture and training hyperparameter search are detailed in Table A1.

Optimal hyperparameters were selected on a subject-specific basis (and montage-specific, where applicable) based on the configuration that yielded the highest average cross-validation accuracy over three training iterations.

**Table A1:** Default hyperparameter settings, sampling functions, and value range for each hyperparameter in the architecture and hyperparameter search.

Hyperparameter	Default	Sampling Function	Value Range
# temporal filters ($F_{1}$)	8	$\left\lfloor 2^{x}\right\rfloor$ for x∈[2,4)	[4, 16)
# spatial filters (*D*)	2	$\left\lfloor 2^{x}\right\rfloor$ for x∈[2,4)	[4, 16)
# pointwise filters ($F_{2}$)	16	$\left\lfloor 2^{x}\right\rfloor$ for x∈[2,4)	[4, 16)
Optimizer	Adam	Uniform	{Adam, SGD, RMSprop}
Learning rate	0.001	$10^{-4x}$ for x∈[0.5,1)	(0.0001, 0.01]
Weight decay	0	$10^{x}$ for x∈[−6,−2)	$\left[10^{-6},10^{-2}\right)$
Early stopping metric	Accuracy	Uniform	{Accuracy, AUROC}

## Appendix B. Baseline CNN Implementation Details

The study compares the proposed method featuring optimized architecture and hyperparameter configurations with various convolutional neural network (CNN)-based deep learning baselines. All models were trained using phase-input montage-specific data filtered between 0.1–12 Hz, chosen for its superior classification performance across experimental setups. Default model training hyperparameters from Table A1 were used to train the CNN baselines.

The DeepConvNet model, initially developed by Schirrmeister et al. [26] for electroencephalography (EEG) decoding, employs five convolutional layers and a softmax classification layer. This architecture has more-than-an-order-of-magnitude-larger number of trainable parameters, making it more data-intensive. Schirrmeister et al. [26] point out this limitation and hypothesize that data augmentation strategies or transfer learning may help address data scarcity issues. Therefore, it is hypothesized that the proposed method will achieve higher within-subject performance in the data-limited regime. Modifications to the architecture include adjusting the temporal convolution kernel size from (1, 5) to (1, 3) to accommodate the lower sampling rate of approximately 40 Hz in our EROS data, compared to the 250 Hz assumed in EEG applications.

Additionally, this comparative study explored default EEGNet architectures from Lawhern et al. [37], which served as the basis for the proposed CNN method. The EEGNet-8,2 and EEGNet-4,2 models feature 8 and 4 temporal filters ($F_{1}$), respectively, with D=2 and $F_{2}=F_{1}*D$. The kernel length was adjusted from the original (1, 64) to (1, 20) to align with the EROS data sampling rate (40 Hz). These models offer a more compact, parameter-efficient alternative to DeepConvNet. However, these baselines were evaluated without subject-specific hyperparameter tuning, a factor expected to impact performance in this study’s subject-specific training and evaluation framework.

## Appendix C. Additional Performance Metrics

Figure A1 illustrates the Receiver Operating Characteristic (ROC) curve and confusion matrix corresponding to the best-performing approach across subjects (best-montage trained on 0.1–12 Hz phase input). Predictions for each subject were obtained by evaluating the best-performing montage classifier, trained using three different random seeds, on the subject’s held-out test trials. Finally, a visualization of the metric was obtained by concatenating single-trial predictions for all subjects and comparing them to their ground truth labels.

Figure A2 and Figure A3 display the subject-specific ROC curves and confusion matrices for each subject’s best-performing montage classifier, respectively. Similarly to the metrics aggregated across subjects, predictions for each subject were obtained by evaluating the subject-specific model on the subject’s held-out test trials, aggregating model predictions across training iterations.

## References

[1] Coupé C., Oh Y.M., Dediu D., Pellegrino F. (2019). Different languages, similar encoding efficiency: Comparable information rates across the human communicative niche. Sci. Adv..

[2] Gratton G., Corballis P.M., Cho E., Fabiani M., Hood D.C. (1995). Shades of gray matter: Noninvasive optical images of human brain reponses during visual stimulation. Psychophysiology.

[3] Gratton G., Chiarelli A.M., Fabiani M. (2017). From brain to blood vessels and back: A noninvasive optical imaging approach. Neurophotonics.

[4] Radhakrishnan H., Vanduffel W., Deng H.P., Ekstrom L., Boas D.A., Franceschini M.A. (2009). Fast optical signal not detected in awake behaving monkeys. NeuroImage.

[5] Medvedev A.V., Kainerstorfer J., Borisov S.V., Barbour R.L., VanMeter J. (2008). Event-related fast optical signal in a rapid object recognition task: Improving detection by the independent component analysis. Brain Res..

[6] Medvedev A.V., Kainerstorfer J.M., Borisov S.V., Gandjbakhche A.H., VanMeter J.W. (2010). Seeing electroencephalogram through the skull: Imaging prefrontal cortex with fast optical signal. J. Biomed. Opt..

[7] Proulx N., Samadani A.A., Chau T. (2018). Online classification of the near-infrared spectroscopy fast optical signal for brain-computer interfaces. Biomed. Phys. Eng. Express.

[8] Baniqued P.L., Low K.A., Fabiani M., Gratton G. (2013). Frontoparietal traffic signals: A fast optical imaging study of preparatory dynamics in response mode switching. J. Cogn. Neurosci..

[9] Ronneberger O., Fischer P., Brox T. (2015). U-Net: Convolutional Networks for Biomedical Image Segmentation. arXiv.

[10] Rajpurkar P., Irvin J., Zhu K., Yang B., Mehta H., Duan T., Ding D.Y., Bagul A., Langlotz C.P., Shpanskaya K.S. (2017). CheXNet: Radiologist-Level Pneumonia Detection on Chest X-Rays with Deep Learning. arXiv.

[11] Faust O., Hagiwara Y., Hong T.J., Lih O.S., Acharya U.R. (2018). Deep learning for healthcare applications based on physiological signals: A review. Comput. Methods Programs Biomed..

[12] Shickel B., Tighe P.J., Bihorac A., Rashidi P. (2017). Deep EHR: A Survey of Recent Advances on Deep Learning Techniques for Electronic Health Record (EHR) Analysis. arXiv.

[13] Rajkomar A., Oren E., Chen K., Dai A.M., Hajaj N., Hardt M., Liu P.J., Liu X., Marcus J., Sun M. (2018). Scalable and accurate deep learning with electronic health records. NPJ Digit. Med..

[14] So C., Kim J.U., Luan H., Park S.U., Kim H., Han S., Kim D., Shin C., il Kim T., Lee W.H. (2022). Epidermal piezoresistive structure with deep learning-assisted data translation. NPJ Flex. Electron..

[15] Gong S., Zhang X., Nguyen X.A., Shi Q., Lin F., Chauhan S., Ge Z., Cheng W. (2023). Hierarchically resistive skins as specific and multimetric on-throat wearable biosensors. Nat. Nanotechnol..

[16] Guo W., Ma Z., Chen Z., Hua H., Wang D., Elhousseini Hilal M., Fu Y., Lu P., Lu J., Zhang Y. (2024). Thin and soft Ti3C2Tx MXene sponge structure for highly sensitive pressure sensor assisted by deep learning. Chem. Eng. J..

[17] Herman P., Prasad G., McGinnity T.M., Coyle D. (2008). Comparative Analysis of Spectral Approaches to Feature Extraction for EEG-Based Motor Imagery Classification. IEEE Trans. Neural Syst. Rehabil. Eng..

[18] Wang B., Wong C.M., Wan F., Mak P.U., Mak P.I., Vai M.I. Comparison of different classification methods for EEG-based brain computer interfaces: A case study. Proceedings of the 2009 International Conference on Information and Automation.

[19] Zhang R., Xu P., Guo L., Zhang Y., Li P., Yao D. (2013). Z-Score Linear Discriminant Analysis for EEG Based Brain-Computer Interfaces. PLoS ONE.

[20] He K., Zhang X., Ren S., Sun J. Deep residual learning for image recognition. Proceedings of the IEEE Conference on Computer Vision and Pattern Recognition.

[21] Lecun Y., Bengio Y., Hinton G. (2015). Deep learning. Nature.

[22] Tabar Y.R., Halici U. (2016). A novel deep learning approach for classification of EEG motor imagery signals. J. Neural Eng..

[23] Olivas-Padilla B.E., Chacon-Murguia M.I. (2019). Classification of multiple motor imagery using deep convolutional neural networks and spatial filters. Appl. Soft Comput..

[24] Miao M., Hu W., Yin H., Zhang K. (2020). Spatial-Frequency Feature Learning and Classification of Motor Imagery EEG Based on Deep Convolution Neural Network. Comput. Math. Methods Med..

[25] Zhang C., Kim Y.K., Eskandarian A. (2021). EEG-inception: An accurate and robust end-to-end neural network for EEG-based motor imagery classification. J. Neural Eng..

[26] Schirrmeister R.T., Springenberg J.T., Fiederer L.D.J., Glasstetter M., Eggensperger K., Tangermann M., Hutter F., Burgard W., Ball T. (2017). Deep learning with convolutional neural networks for EEG decoding and visualization. Hum. Brain Mapp..

[27] Sakhavi S., Guan C., Yan S. Parallel convolutional-linear neural network for motor imagery classification. Proceedings of the 2015 23rd European Signal Processing Conference (EUSIPCO).

[28] Cecotti H., Graser A. (2011). Convolutional Neural Networks for P300 Detection with Application to Brain-Computer Interfaces. IEEE Trans. Pattern Anal. Mach. Intell..

[29] Cecotti H., Eckstein M.P., Giesbrecht B. (2014). Single-Trial Classification of Event-Related Potentials in Rapid Serial Visual Presentation Tasks Using Supervised Spatial Filtering. IEEE Trans. Neural Networks Learn. Syst..

[30] Manor R., Geva A.B. (2015). Convolutional Neural Network for Multi-Category Rapid Serial Visual Presentation BCI. Front. Comput. Neurosci..

[31] Shamwell J., Lee H., Kwon H., Marathe A.R., Lawhern V., Nothwang W., George T., Dutta A.K., Islam M.S. (2016). Single-trial EEG RSVP classification using convolutional neural networks. Proceedings of the Micro- and Nanotechnology Sensors, Systems, and Applications VIII.

[32] Antoniades A., Spyrou L., Took C.C., Sanei S. Deep learning for epileptic intracranial EEG data. Proceedings of the 2016 IEEE 26th International Workshop on Machine Learning for Signal Processing (MLSP).

[33] Liang J., Lu R., Zhang C., Wang F. Predicting Seizures from Electroencephalography Recordings: A Knowledge Transfer Strategy. Proceedings of the 2016 IEEE International Conference on Healthcare Informatics (ICHI).

[34] Mirowski P., Madhavan D., LeCun Y., Kuzniecky R. (2009). Classification of patterns of EEG synchronization for seizure prediction. Clin. Neurophysiol..

[35] Page A., Shea C., Mohsenin T. Wearable seizure detection using convolutional neural networks with transfer learning. Proceedings of the 2016 IEEE International Symposium on Circuits and Systems (ISCAS).

[36] Thodoroff P., Pineau J., Lim A., Doshi-Velez F., Fackler J., Kale D., Wallace B., Wiens J. (2016). Learning Robust Features using Deep Learning for Automatic Seizure Detection. Proceedings of the 1st Machine Learning for Healthcare Conference.

[37] Lawhern V.J., Solon A.J., Waytowich N.R., Gordon S.M., Hung C.P., Lance B.J. (2018). EEGNet: A Compact Convolutional Neural Network for EEG-based Brain-Computer Interfaces. J. Neural Eng..

[38] Chollet F. (2016). Xception: Deep Learning with Depthwise Separable Convolutions. arXiv.

[39] Ogasawara J., Ikenoue S., Yamamoto H., Sato M., Kasuga Y., Mitsukura Y., Ikegaya Y., Yasui M., Tanaka M., Ochiai D. (2021). Deep neural network-based classification of cardiotocograms outperformed conventional algorithms. Sci. Rep..

[40] Wang H., Zhang Q., Lu H., Won D., Yoon S.W. (2019). 3D Medical Image Classification with Depthwise Separable Networks. Procedia Manuf..

[41] Perpetuini D., Günal M., Chiou N., Koyejo S., Mathewson K., Low K.A., Fabiani M., Gratton G., Chiarelli A.M. (2023). Fast Optical Signals for Real-Time Retinotopy and Brain Computer Interface. Bioengineering.

[42] Maclin E.L., Low K.A., Fabiani M., Gratton G. (2007). Improving the signal-to-noise ratio of event-related optical signals. IEEE Eng. Med. Biol. Mag..

[43] Whalen C., Maclin E.L., Fabiani M., Gratton G. (2008). Validation of a method for coregistering scalp recording locations with 3D structural MR images. Hum. Brain Mapp..

[44] Gratton G., Corballis P.M. (1995). Removing the heart from the brain: Compensation for the pulse artifact in the photon migration signal. Psychophysiology.

[45] Gratton G., Brumback C.R., Gordon B.A., Pearson M.A., Low K.A., Fabiani M. (2006). Effects of measurement method, wavelength, and source-detector distance on the fast optical signal. Neuroimage.

[46] Gratton G. (2000). “Opt-cont” and “Opt-3D”: A software suite for the analysis and 3D reconstruction of the event-related optical signal (EROS). Psychophysiology.

[47] Paszke A., Gross S., Massa F., Lerer A., Bradbury J., Chanan G., Killeen T., Lin Z., Gimelshein N., Antiga L. (2019). PyTorch: An Imperative Style, High-Performance Deep Learning Library. arXiv.

[48] Montavon G., Samek W., Müller K.R. (2018). Methods for interpreting and understanding deep neural networks. Digit. Signal Process..

[49] Ancona M., Ceolini E., Öztireli C., Gross M. (2017). Towards better understanding of gradient-based attribution methods for Deep Neural Networks. arXiv.

[50] Shrikumar A., Greenside P., Kundaje A. (2017). Learning Important Features Through Propagating Activation Differences. Int. Conf. Mach. Learn. (ICML).

[51] Ribeiro M.T., Singh S., Guestrin C. (2016). “Why should I trust you?” Explaining the predictions of any classifier. Proc. Acm Sigkdd Int. Conf. Knowl. Discov. Data Min..

[52] Nguyen A., Yosinski J., Clune J. Deep Neural Networks are Easily Fooled: High Confidence Predictions for Unrecognizable Images. Proceedings of the IEEE Computer Society Conference on Computer Vision and Pattern Recognition.

[53] Zeiler M.D., Fergus R. (2013). Visualizing and Understanding Convolutional Networks. Eur. Conf. Comput. Vis. (ECCV).

[54] Baehrens D., Schroeter T., Harmeling S., Kawanabe M., Hansen K., Müller K.R. (2010). How to Explain Individual Classification Decisions. J. Mach. Learn. Res..

[55] Kim S.G., Ashe J., Hendrich K., Ellermann J.M., Merkle H., Ugurbil K., Georgopoulos A.P. (1993). Functional Magnetic Resonance Imaging of Motor Cortex: Hemispheric Asymmetry and Handedness. Science.

[56] Kübler A., Mushahwar V.K., Hochberg L.R., Donoghue J.P. (2006). BCI Meeting 2005 - Workshop on clinical issues and applications. IEEE Trans. Neural Syst. Rehabil. Eng..

[57] Chiarelli A.M., Romani G.L., Merla A. (2014). Fast optical signals in the sensorimotor cortex: General Linear Convolution Model applied to multiple source–detector distance-based data. NeuroImage.

[58] Gratton G., Fabiani M., Friedman D., Franceschini M.A., Fantini S., Corballis P., Gratton E. (1995). Rapid changes of optical parameters in the human brain during a tapping task. J. Cogn. Neurosci..

[59] Morren G., Wolf M., Lemmerling P., Wolf U., Choi J.H., Gratton E., De Lathauwer L., Van Huffel S. (2004). Detection of fast neuronal signals in the motor cortex from functional near infrared spectroscopy measurements using independent component analysis. Med Biol. Eng. Comput..

[60] Wolf M., Wolf U., Choi J.H., Toronov V., Adelina Paunescu L., Michalos A., Gratton E. (2003). Fast cerebral functional signal in the 100-ms range detected in the visual cortex by frequency-domain near-infrared spectrophotometry. Psychophysiology.

[61] Gratton G., Fabiani M. (2003). The event-related optical signal (EROS) in visual cortex: Replicability, consistency, localization, and resolution. Psychophysiology.

